# Pulmonary Hypertension in Underrepresented Minorities: A Narrative Review

**DOI:** 10.3390/jcm13010285

**Published:** 2024-01-04

**Authors:** Johanna Contreras, Jeremy Nussbaum, Peter Cangialosi, Sahityasri Thapi, Ankitha Radakrishnan, Jillian Hall, Prashasthi Ramesh, Maria Giovanna Trivieri, Alejandro Folch Sandoval

**Affiliations:** 1 Division of Heart Failure and Cardiac Transplantation, Icahn School of Medicine at Mount Sinai, New York, NY 10029, USA; jeremy.nussbaum@mountsinai.org (J.N.); peter.cangialosi@mountsinai.org (P.C.); sahityasri.thapi@mountsinai.org (S.T.); ankitha.radakrishnan@mountsinai.org (A.R.); mariagiovanna.trivieri@mountsinai.org (M.G.T.); 2 Department of Medicine, Lewis Katz School of Medicine at Temple University, Philadelphia, PA 19140, USA; jillian.hall@tuhs.temple.edu (J.H.); prashasthi.ramesh@tuhs.temple.edu (P.R.); 3 The Cardiovascular Center, Boston Medical Center, Boston, MA 02118, USA; alejandro.folchsandoval@mountsinai.org

**Keywords:** pulmonary hypertension, social determinants of health, health disparities, clinical trial representation

## Abstract

Minoritized racial and ethnic groups suffer disproportionately from the incidence and morbidity of pulmonary hypertension (PH), as well as its associated cardiovascular, pulmonary, and systemic conditions. These disparities are largely explained by social determinants of health, including access to care, systemic biases, socioeconomic status, and environment. Despite this undue burden, minority patients remain underrepresented in PH research. Steps should be taken to mitigate these disparities, including initiatives to increase research participation, combat inequities in access to care, and improve the treatment of the conditions associated with PH.

## 1. Introduction

Pulmonary hypertension encompasses a group of disorders defined by elevated pulmonary arterial pressures, and it can be caused by the dysfunction of the pulmonary vasculature itself (pulmonary arterial hypertension (PAH)) or, more frequently, secondary to other disease processes. Pulmonary hypertension is a poor prognostic sign and predictor of mortality in many conditions, and pulmonary arterial hypertension is a highly morbid disease [1]. This review will examine the disparities faced by patients with pulmonary hypertension in minoritized racial and ethnic groups with respect to the recognition and diagnosis of the disease, burden of associated diseases, health outcomes, and representation in clinical trials.

It is important to note that race and ethnicity are dynamic, sociocultural constructs that lack any reasonable biological basis [2]. These terms generally refer to groupings based on common ancestry, area of origin, or cultural characteristics, and they have limited utility in medical practice and research. However, they can provide important reference points for social inequities, and they can influence an individual’s environment, access to education, nutrition, political circumstances, and access to healthcare [3]. Racial and ethnic disparities result from complex interactions between patient factors and health system factors, including socioeconomic status, environment, and systemic bias and discrimination [4]. Categorizing patients into racial and ethnic groups may lack a physiologic basis, but it is helpful in studying and mitigating healthcare disparities in underserved and underrepresented populations.

Pulmonary hypertension is defined by increased pulmonary artery pressure, and it is separated into five groups based on etiology. WHO Group 1 PH is known as pulmonary arterial hypertension (PAH) and is due to the dysfunction of the pulmonary vasculature, whether idiopathic or secondary to another disease process [5]. WHO Group 2 PH is due to increased pulmonary artery pressure caused by left-sided heart disease, Group 3 PH is due to lung disease and/or hypoxia, and Group 4 PH is associated with pulmonary artery obstructions (generally thrombo-embolic) [5]. WHO Group 5 PH is defined by unclear and/or multifactorial mechanisms. The recently revised definition of pulmonary hypertension is a mean pulmonary artery pressure (mPAP) > 20 mmHg or a pulmonary vascular resistance (PVR) > 2.0 Wood units; this is a lower threshold than prior and is supported by studies that have assessed the upper limit of normal PA pressure and examined the increased mortality conferred by elevated PVR [5]. This revised definition will hopefully allow patients to be diagnosed and treated earlier in their disease course, improving outcomes and mitigating disparities caused by later presentations in minoritized patients.

## 2. Disparities in Pulmonary Hypertension

### 2.1. Prevalence and Presentation

Pulmonary hypertension is estimated to affect around 1% of the global population, and up to 10% of individuals 65 years or older worldwide [6,7]. Pulmonary hypertension registries worldwide have an underrepresentation of ethnic and racial minority patients, so it is difficult to estimate the prevalence of the disease state in these groups [8].

While the initial presentation of PH can vary from patient to patient, the most commonly reported initial symptom is exertional dyspnea. This is the initial symptom in >50% of patients with PH, and it ultimately occurs in approximately 85% of the PH population [9]. A national registry of patients carrying a diagnosis of primary pulmonary hypertension found that 26% of patients reported fatigue, 22% reported chest pain, 20% reported lower-extremity edema, 17% reported presyncope or syncope, and 12% reported palpitations [10]. Signs and symptoms of PH can be nonspecific and/or overlap with a patient’s underlying conditions, making the diagnosis a challenging one for clinicians. Physical exam findings of PH—such as elevated jugular venous pulsation, cardiac murmurs, edema, and ascites—may be unremarkable early in the disease progression and only present when right ventricular strain and eventual failure emerge [1]. There can be a significant delay in the diagnosis of PH. Approximately 20% of patients in the REVEAL Registry who were diagnosed with pulmonary arterial hypertension reported symptoms for more than 2 years before their disease was recognized. Younger individuals and patients with other common respiratory disorders were most likely to experience delayed PAH recognition [9].

Additionally, disease profiles may vary between racial and ethnic groups. In a cohort analysis of 160 patients from the Johns Hopkins PH program, Black patients were found to have worse functional classes upon presentation, lower exercise capacities, higher levels of natriuretic peptides, more severe hemodynamic metrics, and a trend toward decreased survival [11]. PH associated with sarcoidosis [12] and sickle cell disease [13] is also more frequent in Black individuals. This remains an understudied aspect of PH research.

### 2.2. Health Outcomes

There is limited data regarding the disparities in PAH outcomes, particularly because research studies, registries, and clinical trials have an underrepresentation of minority patients. There have been studies looking at outcomes in PAH with regard to socioeconomic status (SES). In the PAHQuERI study, an employment status of unemployed or receiving disability was found to be a predictor of 3-year mortality [14]. Additionally, other studies have found that lower SES was associated with higher mortality rates and clinical progression after adjusting for age, sex, hemodynamics, functional class, and pharmacotherapies [15,16].

Regarding differences in race, data on minoritized patients and PAH outcomes have been mixed and, notably, there has been an underrepresentation of these patients in much of the current PAH research. One study looking at patients with PAH secondary to systemic sclerosis found that Black patients had worse functional classes upon presentation, worse RV function, more severe hemodynamics, and higher BNPs compared to Americans of European descent, although only 18% of the patients studied were Black [11]. Another study using data from the REVEAL Registry found no difference in survival between Black patients with PAH and patients of other races and ethnicities [17]. There was evidence of decreased survival and poorer right-sided heart function of Black PAH patients compared to White PAH patients; however, that difference was no longer present when adjusted for insurance status [18], suggesting that there is more of an association with access to care and SES. In another study, Hispanic patients with PAH were found to have improved survival, though that difference was not present when adjusted for other social determinants of health [19].

### 2.3. Sex Differences

There have been multiple sex differences described in PAH incidence and outcomes. There is an association of dysfunctional estrogen metabolism in the pathophysiology of PAH; an increase in estrogen metabolites, including 16OHEs and CYP1B1, have been shown to be associated with the development of PAH [20,21]. The incidence of PAH in females decreases at around age 65 years, likely due to menopausal hormone changes with decreases in estrogen and estrogen metabolism [22]. Though premenopausal females are more likely to acquire PAH, they tend to have a better response to treatment and increased survival when compared to males [23,24], as well as higher right ventricular ejection fractions, lower right ventricular masses, and smaller right ventricular volumes [25]. Notably, with more recent restrictions on pregnancy termination, females with PAH who are counseled to terminate their pregnancy but are unable to access these services will likely have increased maternal and fetal morbidity, particularly those of lower SES [8].

## 3. Pulmonary Hypertension Groups and Associated Conditions

### 3.1. Group 1 Pulmonary Hypertension

Group 1 PH can be secondary to a number of conditions, most commonly connective tissue/autoimmune disease (notably scleroderma), congenital heart disease, HIV-associated PH, drug/toxin-induced PH, portal hypertension, and idiopathic PH [26]. Recent attempts to create and categorize the demographics of pulmonary arterial hypertension patients led to the REVEAL Registry. This showed a predominance of PAH in White patients (72.8%), with 12.2% seen in Black patients, 8.9% in Hispanic patients, and 3.3% in Asian/Pacific Islanders. When this was compared with the expected distribution in the population, Hispanic and Asian patients were noted to be underrepresented while Black patients were slightly overrepresented [27]. More stark differences in the demographics were noted when examined according to the etiology of Group 1 PAH. Another recent registry created to identify disparities in the demographics of Group 1 PAH also revealed that Hispanic women were more likely to have congenital heart disease-associated PH and non-Hispanic White patients were more likely to have familial PAH and PH related to drugs or toxins [28]. Connective tissue disease is the most common PAH-associated disease. Systemic sclerosis (SS) makes up approximately 75% of these cases, followed by mixed connective tissue disease at 8% and lupus at 8% [29]. Black individuals have a higher prevalence of SS in America compared to White individuals, with Black patients being twice as likely to have diffuse systemic disease [30]. Furthermore, Black Americans with SS were more likely to have pulmonary hypertension compared to their White counterparts [31]. A 2021 meta-analysis study aimed to investigate the prevalence of lupus in the United States and found that Black women had the highest prevalence, followed by Hispanic individuals, White individuals, and finally Asian/Pacific Islanders [32]. Another study showed there was a higher prevalence of SLE-associated pulmonary hypertension in Black patients (11.5%) compared to White patients (5.9%) [33]. This demonstrates that Black patients are not only more likely to develop systemic sclerosis and lupus, but they are then also more likely to develop PH related to these connective tissue diseases.

### 3.2. Group 2 Pulmonary Hypertension

Group 2 pulmonary hypertension is due to left-sided heart disease and is the most common cause of PH in the United States [34]. While there is a lack of literature on the specific demographics of patients with PH related to left-sided heart disease, there are data on patients with left-sided heart disease. Black individuals have higher rates of heart failure compared to White individuals and tend to have earlier onsets along with increased severity. The MESA study concluded that the highest incidence of CHF was in the Black American population, followed by Hispanic individuals, White individuals, and then Asian individuals [35]. Having a higher likelihood of developing these risk factors can predispose these patients to develop PH in the future. Similarly, the prevalence of hypertension in Black patients is also 3–7× higher than in White patients [36]. A 2018 study aimed to identify racial differences in patients referred for right-heart catheterization and risk of pulmonary hypertension. This study population showed a higher prevalence of combined pre- and post-capillary PH in Black patients compared to White patients, which may suggest that there is a predisposition to develop PH in response to left-sided heart disease [37]. Not only are Black patients more likely to develop risk factors for left-sided heart disease, but they may also be more likely to develop PH from left-sided heart disease compared to their White counterparts.

### 3.3. Group 3 Pulmonary Hypertension

Group 3 pulmonary hypertension is characterized by elevated pressure in the pulmonary circulation due to lung diseases or low oxygen levels [5]. Several studies show that more than 90% of patients with COPD have a mean pulmonary artery pressure higher than 20 mmHg, with roughly 5% of patients with a mean PA pressure higher than 35–40 mmHg [38]. Additionally, the incidence of pulmonary hypertension in idiopathic pulmonary fibrosis is 8–15% at diagnosis, 30–50% in advanced disease, and more than 60% in end-stage disease [39].

Limited research has been performed on health disparities specifically in patients with Group 3 pulmonary hypertension. However, data exist on the increased burden of respiratory diseases in minoritized groups and among the socially disadvantaged. A disproportionate burden of COPD occurs in people of low socioeconomic status due to differences in health behaviors, including tobacco smoking, occupations with exposure to toxins, exposure to indoor biomass fuel, air pollution, and access to healthcare [40]. To make matters worse, lower socioeconomic status is associated with worse COPD outcomes, including higher hospitalization rates, higher mortality rates, and worse quality of life [41].

Despite these disparities, there has been limited enrollment of minority patients in clinical trials specifically targeting Group 3 pulmonary hypertension. The INCREASE trial, which evaluated the safety and efficacy of inhaled treprostinil in patients with pulmonary hypertension due to interstitial lung disease enrolled only 71 Black patients and only 27 Hispanic patients [42]. Other studies did not report the racial or ethnic compositions of their study populations, highlighting a lack of representation of minority patients in these trials [42,43,44,45,46,47,48,49,50]. This lack of diversity in clinical trials not only hinders our understanding of the disease and its treatment in different populations but also perpetuates health disparities.

Another group that warrants attention in the context of health disparities is patients who had severe pneumonia and acute respiratory distress syndrome from coronavirus disease 2019. It has been widely reported that minority patients and those from lower socioeconomic backgrounds had worse outcomes during the COVID-19 pandemic [51]. Although evidence remains limited, recent studies have demonstrated the onset of pulmonary hypertension and right ventricular dysfunction after acute COVID-19 infection, with these hemodynamic effects potentially implicated in the symptoms associated with “post-COVID syndromes” [52,53]. As our understanding of the chronic consequences of COVID-19 infections improves, COVID-19 survivors may become a notable subgroup of Group 3 PH whose management should be considered in a distinct way. As such, it is important to examine and address any potential disparities that may exist in the management of these patients.

### 3.4. Group 4 Pulmonary Hypertension

Group 4 PH is due to pulmonary artery obstructions and is most commonly due to a condition known as chronic thromboembolic pulmonary hypertension (CTEPH). CTEPH is defined as symptomatic pulmonary hypertension with persistent pulmonary perfusion defects despite therapeutic anticoagulation for 3–6 months [5]. The exact epidemiology of CTEPH is unknown, although it is most likely underdiagnosed and therefore undertreated.

The research into disparities in this condition has been limited. A single-center study that retrospectively reviewed all the patients who underwent pulmonary thromboendarterectomy (PTE) from June 2009 to June 2019 found a higher mortality in those patients who had a lower socioeconomic status (assessed using the zip code-linked Distressed Communities Index, a validated, holistic measure of community wellbeing). Of note, race was not associated with a difference in survival in this analysis [54].

In a separate analysis by Chan and colleagues, a retrospective review of 401 consecutive patients who underwent PTE found that women were more likely to receive presurgical oxygen therapy and to have segmental and sub-segmental disease compared to men. Although the pre-operative values were similar, women had higher post-operative pulmonary vascular resistance. Despite this, survival at 10 years was not different between the groups, although females had a higher requirement of targeted pulmonary hypertension therapy compared to males [55].

## 4. Vasodilation Physiology and Treatment Considerations

The pathophysiology of pulmonary hypertension is characterized by endothelial dysfunction, smooth muscle cell remodeling, and issues with the normal vasodilatory pathways [56]. Physiologic vasodilation is mediated by two primary signaling pathways: the cyclic adenosine monophosphate (cAMP) and cyclic guanosine monophosphate (cGMP) pathways. The cAMP pathway is mediated by prostacyclin activation. Prostacyclins or prostacyclin analogues bind to their receptor, leading to decreased intracellular calcium and, in turn, increased vasodilation [57]. By contrast, the cGMP pathway is mediated largely via nitric oxide (NO). NO is produced in the pulmonary vascular endothelium and diffuses into vascular smooth muscle cells, where it binds soluble guanylyl cyclase in order to increase intracellular cGMP [58]. cGMP then phosphorylates cGMP-dependent protein kinase, which leads to a decrease in intracellular calcium, thereby decreasing the myosin light-chain cross-linking and vascular tone [59]. In addition to the cAMP and cGMP pathways, endothelial dysfunction and vasoconstriction via the endothelin receptor pathway are also implicated in pulmonary hypertension pathophysiology [57].

### 4.1. cGMP and Nitric Oxide

Pharmacologic agents for pulmonary hypertension therefore target each of these three different pathways. The cGMP and nitric oxide pathways are primarily targeted by a class of medications called phosphodiesterase inhibitors. These agents work by inhibiting the phosphodiesterase-5-mediated degradation of cGMP, thereby increasing the cGMP levels and downstream vasodilation in the pulmonary vasculature [60]. The first of these agents, sildenafil, was studied in a randomized control trial of 278 patients, the majority of which had WHO Group 1 pulmonary arterial hypertension, and was found to have a significant benefit in improving the Six-Minute Walk Test (6MWT), WHO functional class, and mean pulmonary artery pressure [61]. The second agent in this class, tadalafil, was studied in 405 patients with symptomatic PAH and demonstrated a significant improvement in the 6MWT and quality-of-life measures [62]. In terms of representation, however, both trials enrolled greater than 80% White patients in their study groups [61,62].

Another class of medications to target this pathway is the soluble guanylate cyclase stimulators. These agents work by stimulating soluble guanylate cyclase via a binding site independent of nitric oxide, as well as by stabilizing the nitric oxide binding site to sensitize guanylate cyclase to endogenous NO [63]. Riociguat is the primary option in this drug class and is the only medication currently approved for both Groups 1 and 4 pulmonary hypertension. It was first studied in 443 patients with symptomatic PAH and found to significantly improve the 6MWT, pulmonary vascular resistance (PVR), proBNP levels, and WHO functional class [64]. It was also studied in 261 patients with CTEPH, where it also demonstrated significant benefits in the 6MWT and PVR [65]. While these trials were slightly more diverse compared to the PDE-5 studies, they still enrolled a majority of White patients (61% and 71%) and severely underrepresented Black patients (1% and 3%) in their study groups [64,65].

### 4.2. cAMP and Prostacyclins

Prostacyclin analogues and agonists are the two primary agents used to target the cAMP vasodilation pathway. Prostacyclin is a type of prostaglandin endogenously released by endothelial cells within the pulmonary arteries. Upon binding to its receptors, it activates G-protein, leading to increased intracellular cAMP, thereby triggering protein kinase A and downstream smooth muscle relaxation and pulmonary artery vasodilation [66]. Epoprostenol is an IV prostacyclin analogue that was the first therapy approved for the treatment of PAH, following an 82-patient trial in 1996 demonstrating improvement in the 6MWT, mean PA pressure, and PVR [67]. Treprostinil is an analogue that was first studied in a subcutaneous formulation in a 470-patient trial and found to significantly improve the 6MWT distance [68]. Treprostinil has also shown significant benefits in the exercise capacity, both when studied as a monotherapy [69] and in combination with the endothelin receptor antagonist bosentan [70]. Treprostinil has been most recently studied in a trial of 326 patients with Group 3 pulmonary hypertension, where it showed significant improvement in the 6MWT, making it the only agent specifically approved for Group 3 PAH [42]. Finally, iloprost is an additional inhaled prostacyclin analogue, which has also shown significant benefit in the 6MWT and functional class [71].

Selexipag, by contrast, is an oral agent that acts as an agonist at the prostacyclin receptor. It was studied in a larger trial of 1156 patients and found to increase the time to clinical worsening in PAH [72]. The above trials varied in their degrees of minoritized patient inclusion. Demographic data on race/ethnicity were not reported in the initial epoprostenol trial [67] or the study of iloprost [71]. Greater than 84% of patients in the study of subcutaneous treprostinil were White [68], while the more recent trial of selexipag did recruit across a wide range of geographic areas but did not specifically report data on the patient race/ethnicity [72].

### 4.3. ERA Pathway

The final potential target is the endothelin pathway. Endothelin 1 is an endogenous vasoconstrictor that is overexpressed in the pulmonary vasculatures of patients with PAH [60]. Bosentan and macitentan are both dual endothelin receptor antagonists that have demonstrated significant improvements in the 6MWT and PVR [73,74], as well as in the time to clinical worsening for macitentan specifically [75]. Ambrisentan is a selective endothelin receptor antagonist that has also demonstrated significant improvements in the exercise capacity and time to clinical worsening for patients with PAH [76]. Notably, all of the above trials enrolled majority White patient populations, with less than 6% Black patients in the macitentan and ambrisentan trials [73,75,76].

### 4.4. Treatment Approach

With the variety of medication classes available, multiple large trials and meta-analyses support first-line treatment with a combination therapy of an endothelin-receptor antagonist plus PDE-5 inhibitor compared with monotherapy alone [77]. For example, a 2015 randomized control trial demonstrated the benefit of ambrisentan plus tadalafil as first-line therapy when compared to either monotherapy alone in terms of the time to clinical failure events [78].

However, when patients experience progressive disease despite maximal appropriate medical therapy, and particularly for patients requiring continuous IV prostacyclin therapy, more aggressive interventions, such as lung transplant, can be considered [79]. A patient’s disease progression and risk of short-term mortality can be quantified with a number of risk stratification tools, most commonly the REVEAL risk score [80]. For patients deemed to be appropriate transplant candidates, a double lung transplant is now the standard of care for patients with PAH at most centers [81]. A rapid and significant improvement in the right-sided pressures is typically seen post-transplant [81]; however, patients with PAH have lower 3-month (76%) and 1-year survival rates (71%) compared to patients with pulmonary fibrosis [82]. While it serves as the definitive therapy for PAH, 5-year median survival rates are still just 51.7% [81], further emphasizing the importance of continuing to study and improve upon the existing pharmacologic options.

Finally, the impact of the above pharmacologic treatment modalities is severely limited by the significant cost associated with PH therapies, further propagating disparities in care. While multiple classes of PH-targeted therapies exist, the cost of these drugs is often prohibitive, even for patients with adequate health insurance coverage. Prior studies have estimated the mean average cost per patient with PAH at USD 80,000 per year [8]. A more recent review on the issue from 2016 also found a significant economic burden on patients with PH, primarily related to medication costs, with estimates as high as over USD 11,000 per month for healthcare-related costs [83]. The exorbitant cost of PH therapies is a potential barrier to treatment for nearly all patients and a burden that is no doubt felt more acutely by minoritized patient groups of lower socioeconomic status who are already suffering from poor access to care.

## 5. Representation in Pulmonary Hypertension Trials

The underrepresentation of patients from ethnic and racial minority groups in major cardiac clinical trials is an issue that is not unique to pulmonary hypertension alone. At present, only 15% of patients enrolled in NIH trials are Black [84], and only 30% of the total patients identify as minorities. Unfortunately, the lack of representation of diverse groups in clinical trial enrollment perpetuates a cycle of worsening relationships between healthcare systems and minority patients.

It has been shown in multiple studies that patients in minoritized groups have complicated relationships with healthcare for multiple reasons, including distrust of healthcare professionals, limited healthcare literacy, and language barriers. The lack of representation in trial enrollment of such patients further promotes distrust in the minority community towards clinical trials and findings [85]. Interestingly, some barriers to diverse clinical trial enrollment often overlap with the causes of health disparities amongst minority groups, including distrust of healthcare systems, a lack of access to care, a lack of health professionals from minority groups, and language barriers [86]. It has also been shown that these issues further deepen both conscious and unconscious biases amongst providers regarding minority patients [87]. This vicious cycle ultimately alienates minority groups, decreases patients’ likelihood to participate in trials, and decreases engagement with healthcare systems that practice the evidence-based medicine that is a product of such trials [88].

The inadequate recruitment of minority patients into landmark trials further complicates medical care, due to the limited generalizability of the trial findings to these populations. Major breakthroughs in pulmonary hypertension management are unfortunately not an exception to this predicament. Table 1 illustrates how many major trials in pulmonary hypertension since 2004 have severely under-recruited Black and minority patients, with the vast majority recruiting less than 20% of patients from ethnic minorities. As referenced above, there is a high prevalence of pulmonary hypertension amongst Black patients [28]. However, the current guidelines for the treatment of PH have been heavily influenced by the findings of various clinical trials that are not representative of this population (Table 1). A re-examination of the enrollment practices with respect to race calls into question the generalizability of the past 20 years of scientific discovery in pulmonary hypertension. Whether these findings and guidelines should be applied to all patients irrespective of race and ethnicity remains to be explored. The high prevalence of pulmonary hypertension amongst non-White patients emphasizes the need for a better understanding of how the disease and its treatments may vary between various racial groups.

## 6. Conclusions

**Figure 1:**
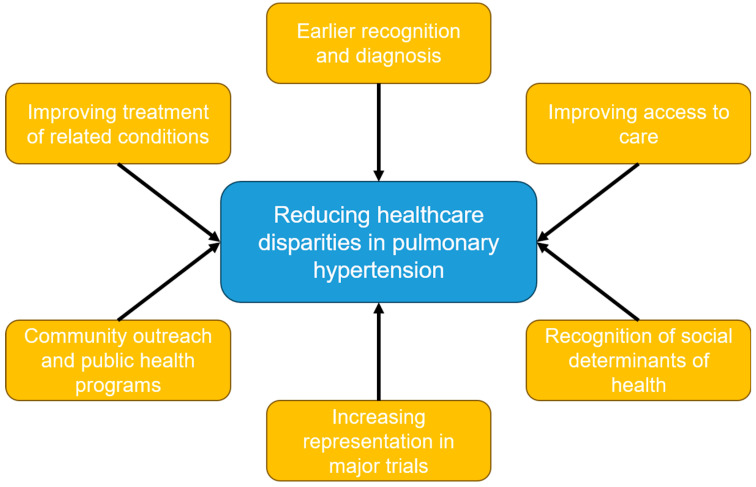
Non-pharmacologic methods to reduce healthcare disparities in pulmonary hypertension.

Pulmonary hypertension represents a heterogenous group of conditions that all have a high degree of morbidity and mortality. Racial and ethnic minority patients suffer from an increased burden of pulmonary hypertension and its associated conditions, such as connective tissue diseases (including systemic sclerosis and lupus), left-sided heart disease, and respiratory diseases such as COPD and interstitial lung disease. Many of these disparities are explained by the known social determinants of health, such as access to high-quality medical care, health literacy, the environment, socioeconomic status, systemic biases, and a mistrust of and lack of engagement in the healthcare system. Despite this undue burden, racial and ethnic minority patients remain vastly underrepresented in trials studying pulmonary hypertension. There remain many questions to be answered regarding the healthcare disparities in PH, and there are several avenues to explore to mitigate these disparities (Figure 1). This may be accomplished by promoting more diverse research representation by enrolling more minority patients in clinical trials and incorporating recruitment efforts in smaller, more diverse communities. Another area to explore could include community outreach by advocating for the creation of more community PH programs as well as public health programs that can help increase access to general medical services. Fostering ethnic and racial diversity among PH healthcare providers, as well as increasing awareness of social determinants of health, may also help reduce these disparities. Lastly, the early diagnosis and treatment of PH and its associated conditions should be pursued as much as possible to reduce the burden suffered by underserved and underrepresented minority patient groups.

## Figures and Tables

**Table 1:** Major trials in pulmonary hypertension and recruitment of minority patients.

Trial Name	Major Conclusions	Minority Recruitment
Channick et al. [73]	Bosentan improves exercise capacity and hemodynamics in patients with pulmonary hypertension.	<18% Black patients
Rubin et al. [74]	Bosentan is an effective medication in patients with pulmonary arterial hypertension.	>77% White patients
BREATHE-2 [89]	Trial suggested a trend towards hemodynamic or clinical improvement in patients with pulmonary arterial hypertension who were treated with a combination of epoprostenol and bosentan.	<10% Black patients
SUPER-1 [61]	In patients with pulmonary arterial hypertension, sildenafil improves exercise capacity, WHO functional class, and hemodynamics.	<10% non-White patients >80% White patients
ARIES 1 and 2 [76]	Ambrisentan improves exercise capacity in pulmonary arterial hypertension.	0–6% Black patients 66–92% White patients
SERAPHIN [90]	Macitentan reduced morbidity and mortality amongst patients with pulmonary arterial hypertension.	2.6% Black patients 54.5% White patients
CHEST1 [65] and CHEST2 [91]	Riociguat improved exercise capacity and pulmonary vascular resistance in patients with chronic thromboembolic pulmonary hypertension.	3% Black patients 71% White patients
GRIPHON [72]	In patients with pulmonary arterial hypertension, treatment with selexipag led to a lower risk of death and pulmonary hypertension complication.	<10% enrollment in Latin America Predominantly enrolled in Europe
AMBITION [78]	Amongst those with pulmonary arterial hypertension, combination therapy with ambrisentan and tadalafil led to less risk of composite death, hospitalization for pulmonary arterial hypertension, disease progression, and unsatisfactory long-term clinical response than monotherapy with either agent.	<15% non-White patients
VICTORIA [92]	Amongst patients with a high risk of heart failure, those who received vericiguat had less risk of death due to cardiovascular causes or heart failure hospitalizations than those who received a placebo.	4.9% Black patients 64% White patients
PULSAR [93]	Amongst patients receiving background therapy for pulmonary arterial hypertension, treatment with sotatercept resulted in a reduction in pulmonary vascular resistance.	4% Black patients 92% White patients
INCREASE [42]	In patients with pulmonary hypertension secondary to interstitial lung disease, inhaled treprostinil improved exercise capacity.	71 Black patients 238 White patients

## Data Availability

Data sharing not applicable.
